# The skeletal muscle proteomic determinants of neuromuscular function in young and older women following 8 weeks of resistance training

**DOI:** 10.1113/EP092328

**Published:** 2024-12-11

**Authors:** Mary O'Leary, Elsa Greed, Jack Pritchard, Lauren Struszczak, Esra Bozbaş, Joanna Bowtell

**Affiliations:** ^1^ Faculty of Health and Life Sciences, Department of Public Health and Sport Sciences University of Exeter Exeter Devon UK; ^2^ School of Sport, Exercise and Rehabilitation Sciences University of Birmingham Edgbaston Birmingham UK

**Keywords:** exercise, proteomics, resistance training, skeletal muscle, strength

## Abstract

Resistance training (RT) is the gold standard intervention for ameliorating sarcopenia. Outstanding mechanistic questions remain regarding the malleability of the molecular determinants of skeletal muscle function in older age. Discovery of proteomics can expand such knowledge. We aimed to compare the effect of RT on the skeletal muscle proteome and neuromuscular function (NMF) in older and younger women. Seven young (22 ± 6 years) and eight older (63 ± 5 years) women completed 8 weeks’ leg RT. Pre‐ and post‐training, measures of leg and handgrip strength, NMF and vastus lateralis (VL) biopsies were obtained. Tandem‐mass‐tagged skeletal muscle proteomic analyses were performed. Data were analysed using differential expression and weighted gene co‐expression network approaches. Proteins related to skeletal muscle contraction were lower in older skeletal muscle; this was not normalised by RT. Following RT, older women had higher expression of VL mitochondrial biogenesis proteins compared to the young, a reversal of pre‐training observations. Seventy proteins were differentially expressed between age groups. VL expression of these proteins in older women was consistently and significantly associated with poorer leg strength/NMF. Conversely, VL expression of these proteins in older women was often associated with greater handgrip strength. This study has identified important differences in the molecular responses of young and old skeletal muscle to RT. We have demonstrated their close relationship with skeletal muscle function. Proteins that are refractory to RT may represent targets to ameliorate sarcopenia. We have described a ‘proteomic‐function’ relationship that appears to be muscle‐specific. Future research should further unpick these complex relationships.

## Abstract (highlights)

- **What is the central question of this study?**
  How does lower limb RT affect the skeletal muscle proteome and NMF in older versus younger women?
- **What is the main finding and its importance?**
  RT increased proteins related to mitochondrial biogenesis in older women, reversing pre‐training deficits, but did not normalise expression of proteins related to skeletal muscle contraction and the extracellular matrix. Individual proteins were identified within this aged proteome that are associated with poorer leg strength and NMF. Some appear to be training resistant.

## 1 INTRODUCTION

Skeletal muscle mass declines progressively starting in middle age, diminishing at a rate ranging between 3% and 8% per decade (Iii et al., 2000). Sarcopenia represents an age‐related decline in both muscle mass and muscle function that has reached a significant threshold due to this ongoing deterioration (Cruz‐Jentoft et al., 2019). Globally, approximately 10% of individuals aged over 65 years meet diagnostic criteria for sarcopenia (Yuan & Larsson, 2023). Beyond the adult peak in skeletal muscle mass, typically in the mid‐30s, all adults exist somewhere on a ‘sarcopenic decline spectrum’ (Metter et al., 1999). More profound declines in skeletal muscle mass and function have been associated with an elevated risk of cardiovascular, metabolic, cognitive and musculoskeletal conditions, alongside lower quality of life scores and a heightened incidence of falls and fractures (Yuan & Larsson, 2023). The skeletal muscle fibre‐level changes that underpin a decline in skeletal muscle function are well understood. Skeletal muscle fibre size and numbers are decreased in older age, with type II fibres being most profoundly affected (Lexell, 1995; Lexell & Taylor, 1991; Lexell et al., 1988). Aged skeletal muscle fibres display reduced shortening velocity and force production, with declines in myosin heavy chain content having long been implicated in these changes (D'Antona et al., 2003; Frontera et al., 2000; Larsson et al., 1997; Power et al., 2016). The cellular and molecular mechanisms that underpin these functional changes are manifold, interdependent and still incompletely understood. These include chronic local and systemic inflammation, obesity, insulin resistance, altered satellite cell function, anabolic resistance, altered ion handling, epigenetic changes, mitochondrial dysfunction, altered neuromuscular processes and lower motor neuron loss (Antoun et al., 2022; Pascual‐Fernández et al., 2020; Tan et al., 2020).

Physical activity and in particular resistance training (RT) remains the gold‐standard intervention for the prevention and reversal of age‐related skeletal muscle functional decline. RT interventions consistently enhance skeletal muscle mass and strength in older adults (da Silva Gonçalves et al., 2023; Lu et al., 2021; Rocha et al., 2024). Further, RT can reverse some of the known molecular events that have been implicated in sarcopenia, for example, satellite cell numbers (Dewi et al., 2023) and mitochondrial content (Lippi et al., 2022), but many outstanding mechanistic questions remain.

The emergence of multiple proteomics bioanalytical approaches (e.g., two‐dimensional gel electrophoresis, label‐free mass spectrometric analyses and labelled stable isotope mass spectrometric approaches), has allowed for unprecedented insights into protein diversity and function, including in tissues like skeletal muscle. Further, free bioinformatics analysis tools such as The Database for Annotation, Visualization and Integrated Discovery (DAVID) and Reactome, along with curated protein pathway/family databases (e.g., KEGG, Reactome, Wikipathways) have allowed researchers to explore these data in greater depth (Kanehisa & Goto, 2000; Kanehisa et al., 2023; Milacic et al., 2024; Sherman et al., 2022).

Early proteomic analyses of aged human skeletal muscle found age‐related decreases in proteins related to myofilaments, the cytoskeleton, calcium signalling and ion handling, energy metabolism, oxidation, and stress responses (Brocca et al., 2017; Gueugneau et al., 2014; Staunton et al., 2012; Théron et al., 2014). Recent work using more advanced approaches has confirmed and expanded upon these early observations. Ubaida‐Mohien, Lyashkov et al. (2019) used a tandem mass tagged (TMT), liquid chromatography with tandem mass spectrometry (LC‐MS/MS) approach to examine the vastus lateralis (VL) proteome in individuals aged 20–87 years. A linear mixed regression model was used to examine the effect of age on protein expression, with biological themes determined via manual annotation of proteins using Uniprot, GO and PANTHER databases. Proteins related to energy metabolism, mitochondria, muscle contraction and sarcomere stabilisation were under‐represented in older age, with myosin‐binding protein H and microfibril‐associated glycoprotein 4 being most prominently decreased. Proteins related to the immune system, proteostasis and alternative splicing were over‐represented. A similar study by the same group used a linear mixed regression model to examine relationships between self‐reported physical activity and skeletal muscle protein expression after adjusting for age (Ubaida‐Mohien, Gonzalez‐Freire et al., 2019). Individuals reporting higher activity levels had increased levels of proteins related to energy metabolism, mitochondria, muscle contraction and genome maintenance, suggesting an ‘age protective effect’ of exercise. To our knowledge, only three studies have addressed the effect of a RT intervention on the older skeletal muscle proteome; all had methodological limitations that demand their work be further developed. One was a pilot study which performed limited proteomic analyses (Vann et al., 2020). In another, skeletal muscle from older and younger adults undergoing 20 weeks of RT was analysed using an isobaric tags for relative and absolute quantification (iTRAQ) labelling approach (Deane et al., 2023). Skeletal muscle samples were pooled by age group prior to analysis, which limited the quantitative value of the study. Further, only 164 proteins could be used for downstream bioinformatics. Results suggested that RT increased proteins related to mitochondrial function and metabolism but did not alter the well‐documented age‐related reduction in cytoskeletal proteins. Finally, 12 weeks of RT in younger an older adults demonstrated differential VL expression of mitochondrial ribosomal proteins prior to training (33 lower expressed and 14 higher expressed in older adults). Both younger and older adults tended to increase expression of these proteins in response to RT, However, no pathway analyses were performed for proteomics data. No direct older versus younger comparison is presented for post‐training VL. The total number of proteins detected in samples is not detailed and it is unclear whether changes in non‐mitochondrial proteome were sought or simply not present in the VL proteomes described (Robinson et al., 2017).

Therefore, we aimed to use our established TMT LC‐MS/MS skeletal muscle proteomics approach—which typically detects c. 3000 proteins in all samples—to examine the effect of RT on the skeletal muscle proteome in older women compared to young controls. Uniquely, we aimed to explore relationships between the aged skeletal muscle proteome and measures of lower limb neuromuscular function (NMF) taken both before and after RT. We hypothesised that proteins that are differentially expressed in older age would display strong correlations with measures of skeletal muscle function.

## 2 METHODS

### 2.1 Ethical approval

Two double‐blind, placebo‐controlled studies of a herbal supplement were approved by the University of Exeter's Sport and Health Sciences Research Ethics Committee (Refs: v140922, 21‐10‐20‐B‐06). The study conformed to the standards set by the *Declaration of Helsinki*, except for registration in a database. Young (18–35 years) and older (≥55 years) women were recruited through social media adverts. Thirty‐nine women (17 young, 22 older) completed these studies. Skeletal muscle biopsies were available from seven young and eight older women randomised to the placebo groups (magnesium stearate 1000 mg/day) in these studies. These samples were used for proteomic analyses presented here.

Exclusion criteria included: <1 year post‐menopause (older women), participants with self‐reported or diagnosed menstrual irregularities (amenorrhea, anovulation, oligomenorrhea; young women), regular lower limb RT (twice weekly or greater), lower limb musculoskeletal injury, recent NSAID or aspirin use, lidocaine allergy, and any medical condition preventing RT. All participants passed a Physical Activity Readiness Questionnaire.

### 2.2 Study overview

Procedures are detailed in the sections that follow. Briefly, the study protocol included laboratory visits and an 8‐week leg RT programme. During visit 1, participants completed a health screening questionnaire, provided informed consent in writing, and had their height and weight measured. They were familiarised with handgrip strength (HGS) and NMF testing protocols. Equipment settings were customised and recorded for future visits. Prior to all subsequent non‐training visits, participants abstained from caffeine, alcohol and strenuous exercise for 48 h. At visit 2, participants underwent a standardised warm‐up and familiarisation protocol for knee extension (KE) and leg press (LP) exercises, followed by measurement of one repetition maximum (1RM) for KE, LP and HGS. The interval between visits 2 and 3 ranged from 2 to 7 days. Prior to visit 3, participants recorded a 3‐day food diary. NMF tests were conducted, along with blood sampling and VL skeletal muscle biopsy. Participants then consumed two magnesium stearate capsules daily (1000 mg/day) during a twice‐weekly, 8‐week lower limb RT intervention. At the final ‘training’ session, KE 1RM, LP 1RM and HGS were measured as before. Prior to visit 4, participants replicated their 3‐day food diary intake. There was a 48–72 h interval between visits 3 and 4. At visit 4, participants repeated visit 3 procedures. Compliance was assessed by counting remaining capsules.

### 2.3 Measurement of leg strength

Leg strength was assessed by determining 1RM on KE (Life Fitness, Rosemont, IL, USA) and LP press (Body‐Solid, Forest Park, IL, USA) machines, respectively. Participants completed a standardised warm‐up protocol and then attempted 1RM lifts, with weights increased until failure. A rest interval of 3 min was employed between attempts (Grgic et al., 2020).

### 2.4 Measurement of handgrip strength

HGS was assessed using a handgrip dynamometer (Takei 5001 Grip Dynamometer Analogue, Takai Scientific Instruments Co., Ltd, Niigata City, Japan), following an adapted version of the Southampton protocol (Roberts et al., 2011). Participants performed three maximum isometric tests for each hand, alternating between hands. The best score was used for data analysis. HGS was measured during visit 2 and at participants final 1RM testing session.

### 2.5 Lower limb resistance training

Twice weekly, participants completed KE and LP exercises. Each training session began with a two‐set KE warm‐up, consisting of one set of eight repetitions at 50% of the previous session's final weight, and one set consisting of three repetitions at 70% of that weight, separated by a 2‐min rest interval. Following the warm‐up, participants performed exhaustive sets on both machines, beginning with KE. For weeks 1–3, two sets of 12–15 repetitions were desired; in weeks 4–6, three sets of 10–12 were desired; and in weeks 7–8, four sets with an 8–10 repetition range were desired. The starting weight for participants’ initial training session was 70% 1RM. This weight was adjusted if participants had ease or trouble completing the 12–15 repetitions in the first week. The weight lifted was increased throughout the training programme if participants achieved two repetitions above the desired range for two consecutive sets. KE was increased in 3.5 kg increments and the LP in 2.5 kg increments. Throughout each set, participants received consistent verbal encouragement and feedback on technique and ranges of motion.

### 2.6 Neuromuscular function testing

In visits 1 (familiarisation), 3 (pre‐training) and 4 (post‐training), electromyography (EMG) was used to measure electrical activity in the VL and biceps femoris (BF) during voluntary contractions and involuntary contractions evoked by peripheral nerve stimulation (PNS) in the resting and maximally contracting knee extensors. Participants were positioned on a leg‐extension dynamometer with their non‐dominant leg secured to prevent a contribution to force production. EMG and femoral nerve stimulation electrodes were positioned and the stimulation intensity which elicited *M*_max_ was determined as previously described elsewhere (Bowtell et al., 2018). Participants then received five single‐pulse femoral nerve stimulations (130% *M*_max_) to ensure supramaximal stimulation. Following this, 10 alternating non‐stimulated (*n* = 5) and stimulated (*n* = 5) maximal voluntary contractions (MVC) were performed interspersed with 1 min rest. Vocal encouragement was provided to encourage maximal efforts during each 3 s effort. During stimulated MVCs, single‐pulse femoral nerve stimulation (130% *M*_max_) was delivered during (superimposed twitch) and 2 s (potentiated twitch) after the MVC. The EMG signals were processed and data analysed as previously described (Bowtell et al., 2018).

### 2.7 Vastus lateralis biopsy

Following NMF testing, a biopsy site was prepared on the lateral aspect of the dominant leg, overlying the VL (Wangdi et al., 2022). A skeletal muscle sample of around 150 mg was obtained via the suction‐modified Bergstrom method (Shanely et al., 2014). Muscle tissue samples were immediately frozen in liquid nitrogen and stored at –80°C until analysis.

### 2.8 Proteomics

Skeletal muscle samples (∼15 mg) from 15 participants (7 young, 8 older) were prepared in radioimmunoprecipitation assay (RIPA) buffer in the presence of protease and phosphatase inhibitors (Pierce A32961 Protease and Phosphatase Inhibitor EDTA‐free mini tablet, Thermo Fisher Scientific, Waltham, MA, USA). Samples were homogenised for 1 min using a bead homogeniser (Speedmill Plus, Analytik Jena AG, Jena, Germany). Samples were transferred to microcentrifuge tubes and vortexed for 1 min before incubation on ice for 30 min, with occasional vortexing. Samples were centrifuged for 10 min at 8000 *g* at 4°C. The supernatant was removed to a clean microcentrifuge tube and the pellet was discarded. Protein concentrations were determined by the bicinchoninic acid (BCA) assay (Pierce 23,225 BCA Protein Assay Kit, Thermo Fisher Scientific) according to the manufacturer's instructions.

Aliquots of 50 µg of each sample were treated with trypsin (1.25 µg trypsin; 37°C, overnight), labelled with Tandem Mass Tag (TMTpro) reagents according to the manufacturer's protocol (Thermo Fisher Scientific). The labelled samples were pooled. These 30 samples (15 pre‐training, 15 post‐training) were analysed alongside 26 others that were donated by participants taking part in an active supplement condition that formed part of the wider trial. The 56 samples were analysed in four 15Plex experiments, each containing 14 experimental samples (7 ‘pre and post’ training pairs) plus a common ‘reference’ sample.

An aliquot of 200 µg of the pooled sample was desalted using a SepPak cartridge according to the manufacturer's instructions (Waters). Eluate from the SepPak cartridge was evaporated to dryness and resuspended in buffer A (20 mM ammonium hydroxide, pH 10) prior to fractionation by high pH reversed‐phase chromatography using an Ultimate 3000 liquid chromatography system (Thermo Fisher Scientific). In brief, the sample was loaded onto an XBridge BEH C18 column (130 Å, 3.5 µm, 2.1 mm × 150 mm, Waters) in buffer A and peptides eluted with an increasing gradient of buffer B (20 mM ammonium hydroxide in acetonitrile, pH 10) from 0% to 95% over 60 min. The resulting fractions (20 in total) were evaporated to dryness and resuspended in 1% formic acid prior to analysis by nano‐LC‐MS/MS using an Orbitrap Fusion Lumos mass spectrometer (Thermo Scientific).

High pH reversed phase fractions were further fractionated using an Ultimate 3000 nano‐LC system in line with an Orbitrap Fusion Lumos mass spectrometer (Thermo Scientific). In brief, peptides in 1% (v/v) formic acid were injected onto an Acclaim PepMap C18 nano‐trap column (Thermo Scientific). After washing with 0.5% (v/v) acetonitrile 0.1% (v/v) formic acid, peptides were resolved on a 250 mm × 75 µm Acclaim PepMap C18 reverse phase analytical column (Thermo Scientific) over a 150 min organic gradient, using seven gradient segments (1–6% solvent B over 1 min, 6–15% B over 58 min, 15–32% B over 58 min, 32–40% B over 5 min, 40–90% B over 1 min, held at 90% B for 6 min and then reduced to 1% B over 1 min) with a flow rate of 300 nl min^−1^. Solvent A was 0.1% formic acid and solvent B was aqueous 80% acetonitrile in 0.1% formic acid. Peptides were ionized by nano‐electrospray ionization at 2.0 kV using a stainless steel emitter with an internal diameter of 30 µm (Thermo Scientific) and a capillary temperature of 300°C.

All spectra were acquired using an Orbitrap Fusion Lumos mass spectrometer controlled by Xcalibur 3.0 software (Thermo Scientific) and operated in data‐dependent acquisition mode using an SPS‐MS3 workflow. FTMS1 spectra were collected at a resolution of 120,000, with an automatic gain control (AGC) target of 200,000 and a max injection time of 50 ms. Precursors were filtered with an intensity threshold of 5000, according to charge state (to include charge states 2–7) and with monoisotopic peak determination set to Peptide. Previously interrogated precursors were excluded using a dynamic window (60 s, ±10 ppm). The MS2 precursors were isolated with a quadrupole isolation window of 0.7 *m*/*z*. ITMS2 spectra were collected with an AGC target of 10,000, max injection time of 70 ms and collision‐induced dissociation collision energy of 35%.

For FTMS3 analysis, the Orbitrap was operated at 50,000 resolution with an AGC target of 50,000 and a max injection time of 105 ms. Precursors were fragmented by high energy collision dissociation (HCD) at a normalised collision energy of 60% to ensure maximal TMT reporter ion yield. Synchronous precursor selection (SPS) was enabled to include up to 10 MS2 fragment ions in the FTMS3 scan.

The raw data files were processed and quantified using Proteome Discoverer software v2.4 (Thermo Scientific) and searched against the UniProt Human database (downloaded January 2022: 178,486 entries) using the SEQUEST HT algorithm. Peptide precursor mass tolerance was set at 10 ppm, and MS/MS tolerance was set at 0.6 Da. Search criteria included oxidation of methionine (+15.995 Da), acetylation of the protein N‐terminus (+42.011 Da) and methionine loss plus acetylation of the protein N‐terminus (−89.03 Da) as variable modifications and carbamidomethylation of cysteine (+57.0214 Da) and the addition of the TMTpro mass tag (+304.207 Da) to peptide N‐termini and lysine as fixed modifications. Searches were performed with full tryptic digestion, and a maximum of 2 missed cleavages were allowed. The reverse database search option was enabled and all data were filtered to satisfy false discovery rate (FDR) of 5%.

Data were analysed at the University of Exeter. Data were normalised to the total peptide amount in each sample and scaled using a pooled ‘reference’ sample common to both TMT experiments to facilitate the comparison of protein levels between experiments. Data were log_2_ transformed prior to analysis. Data were further filtered to satisfy an FDR of 1% and to include only proteins that were detected in all samples (2912 proteins). Reactome (V8, online version; https://reactome.org/) was used to perform differential gene expression analysis using the ‘Correlation Adjusted MEan RAnk gene set test’ (CAMERA) algorithm. The FDR (Benjamini–Hochberg‐adjusted *P*‐value < 0.05) and log_2_ fold change (log_2_FC) values were calculated by Reactome and are reported for both individual proteins and pathways. Individual protein expression was considered to be significantly altered if the FDR was <0.05. All proteins with a non‐log adjusted fold change >1.2 or <0.8 were considered to warrant presentation. Lists of differentially expressed proteins were uploaded to DAVID (https://david.ncifcrf.gov/), to interrogate differentially expressed KEGG and GO biological process terms. A background protein list consisting of proteins that were detected in all samples was used. Principal component analysis (PCA) was performed on log_2_ transformed proteomics data in R using the prcomp function of the package ‘stats’ (v3.6.2). Protein networks were visualised using in Cytoscape v3.10.0 using stringApp v2.0.1 (Reimand et al., 2019). The full STRING network was used with a high confidence threshold of 0.7. Log_2_FC values (older vs. young participants) were used to style the network, indicating expression of individual proteins. Network clustering was performed using the Markov clustering (MCL) implementation in the clusterMaker2 Cytoscape app; the inflation value was set to 4.0.

### 2.9 Protein correlation and weighted gene co‐expression network analysis

Weighted gene co‐expression network analysis (WGCNA) was performed to identify co‐expressed protein modules in the whole dataset (R version 4.3.2, WGCNA version 1.72‐1; a power parameter with a signed R2 above 0.80 was chosen with a soft threshold of 10 used for WGCNA). Module eigengenes were correlated with physiological traits using WGCNA's weighted Pearson correlation. Student's asymptotic *P*‐value was calculated for these correlations. Correlations between differentially expressed proteins (older vs. young) and physiological traits were calculated using the same approach. To facilitate these comparisons, KE and LP training loads were calculated for each participant from weekly total repetitions completed multiplied by total weight lifted (kg) for each of the eight training weeks. The DescTools (v0.99.54, R version 4.3.2) area under the curve (AUC) function (trapezoid interpolation) was then used to calculate a training load value for each participant.

### 2.10 Functional data analyses

Histograms of functional data were used to check for normality and any outliers. The data were normally distributed and Student's paired samples *t*‐test was used to assess differences between pre‐ and post‐training strength and NMF variables; an unpaired *t*‐test was used to assess differences between age‐cohort baseline physiological parameters. Statistical analyses were performed in SPSS version 23 (IBM Corp., Armonk, NY, USA). *P* < 0.05 was set as the significance threshold. Data are presented as means ± SD.

## 3 RESULTS

### 3.1 Participants, compliance and adverse events

Data obtained from seven young (22.4 ± 5.7 years, BMI 23.0 ± 2.2 kg/m^2^) and eight older (63.4 ± 4.6 years, BMI 24.6 ± 3.7 kg/m^2^) women were used for analyses. Participants reported no serious adverse events during the study. No participant missed more than 3/16 training sessions. Mean training session compliance was 94%.

### 3.2 Effect of resistance training on skeletal muscle strength and neuromuscular function

Older women had a significantly lower KE 1RM and knee extensor excitability (amplitude rest) than their younger counterparts at baseline, but better HGS (Table 1). Leg press 1RM did not differ between age cohorts at baseline. Eight weeks RT increased LP (young 136%, *P* = 0.0104; older 155%, *P* = 0.00401) and KE (young 128% *P* < 0.0001; older 118%, *P* < 0.0001) 1RM (Table 1). VL EMG amplitude was significantly increased by training in young, but not older participants (young pre 3.58 ± 0.91 mV, post 4.78 ± 1 mV, *P* = 0.0235; older pre 2.58 ± 0.39 mV, post 2.65 ± 0.69 mV, *P* = 0.646). No other NMF measure was significantly altered by training in this small cohort. Pearson correlations of age (as a continuous variable) with strength and NMF outcomes indicated that age was negatively correlated with KE 1RM (*r* = 0.60, *P* < 0.001) and measures of excitability—amplitude rest (*r* = −0.62, *P* = 0.000922) amplitude MVC (*r* = −0.51, *P* = 0.00575) and amplitude potentiated (*r* = −0.58, *P* = 0.00110). Age was not correlated with any LP measure, any training load measure, peak twitch measures or HGS.

**TABLE 1:** Effect of resistance training on skeletal muscle strength and neuromuscular function in young (*n* = 7) and older (*n* = 8) women.

	Young			Older			Young versus older
	Pre	Post	*P*, Pre versus Post	Pre	Post	*P*, Pre versus Post	*P*, baseline
**Leg press 1RM (N)**	117.8 ± 69.4	160.1 ± 88.1	0.0104^*^	109.6 ± 21.5	167.7 ± 44.0	0.00401^*^	0.756
**Knee extension 1RM (N)**	73 ± 10.6	93.2 ± 14.0	<0.0001^*^	60.0 ± 10.1	70.9 ± 10.7	<0.0001^*^	0.0252^*^
**Handgrip strength (kg)**	23.0 ± 1.6	25.7 ± 2.6	0.00560^*^	26.3 ± 3.0	28.0 ± 4.0	0.294	0.0215^*^
**M‐wave amp rest (mV)**	3.6 ± 0.91	4.78 ± 1	0.0235^*^	2.6 ± 0.39	2.7 ± 0.69	0.646	0.0140^*^
**M‐wave amp MVC (mV)**	3.7 ± 0.8	4.9 ± 1.2	0.0184^*^	3.1 ± 0.6	3.2 ± 0.6	0.725	0.144
**M‐wave amp pot (mV)**	3.6 ± 1.1	4.6 ± 0.9	0.0291^*^	2.9 ± 0.5	2.7 ± 0.7	0.469	0.0911
**Peak twitch rest (N)**	111.08 ± 31.7	107.73 ± 32.1	0.368	85.7 ± 25.7	92.2 ± 30.7	0.218	0.110
**Peak twitch MVC (N)**	14.9 ± 13.4	8.9 ± 7.8	0.150	10.7 ± 5.7	11.3 ± 7.5	0.781	0.428
**Peak twitch pot (N)**	173.1 ± 42.7	165.8 ± 37.1	0.258	140.7 ± 30.0	152.5 ± 31.5	0.0606	0.109

Data are means ± SD. *P < 0.05. Abbreviations: 1RM, one repetition maximum; amp, amplitude; MVC, maximum voluntary contraction; pot, potentiated.

### 3.3 Effect of training on skeletal muscle proteome in non‐resistance trained women

First, the effect of RT on the skeletal muscle proteome was explored in each age cohort separately. No individual protein was significantly (FDR < 0.05 following Benjamini–Hochberg correction for multiple comparisons) altered post‐training in either cohort. Therefore, differential pathway analysis was performed on these data in Reactome.

**FIGURE 1:**
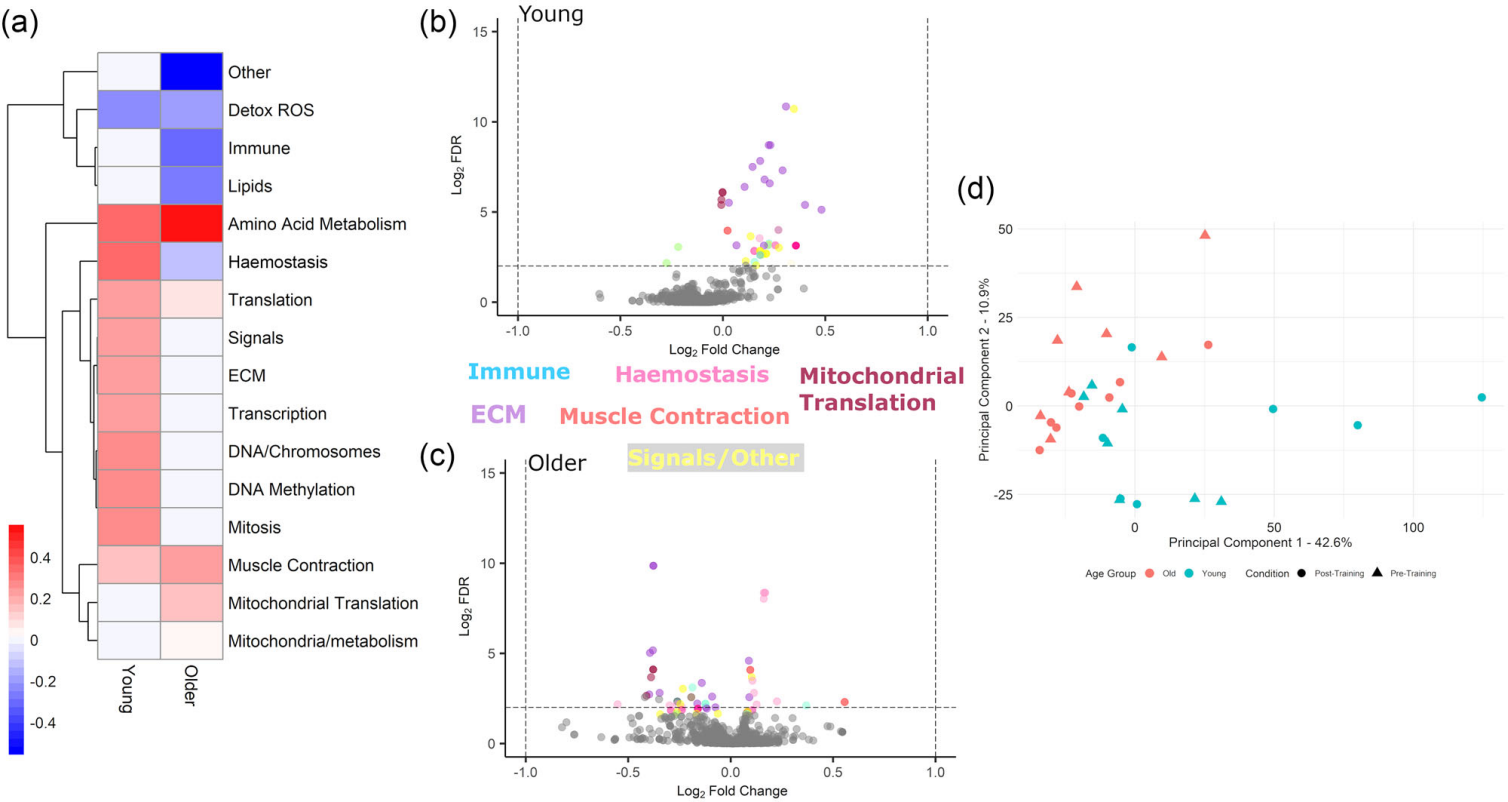
Visualisation of skeletal muscle protein pathway changes following resistance training in young (*n* = 7) and older (*n* = 8) women. (a) Reactome pathway themes significantly altered by resistance training in younger or older women. A blue−white−red gradient was styled using the mean log_2_ fold change value for each theme compared to pre‐training values. White: non‐significant change (false discovery rate < 0.01). (b) Reactome pathway changes in young skeletal muscle following resistance training. Significantly altered subpathways are colour‐coded by theme (see below). (c) Reactome pathway changes in older skeletal muscle following resistance training. Significantly altered subpathways are colour‐coded by theme (see below). Additional themes: lipids: orange; mitochondria/metabolism: dark blue; transcription: aquamarine; detox reactive oxygen species: light green; DNA/chromosomes: light pink; mitosis: chocolate; amino acid metabolism: beige. For additional pathway details, including ‘other’ pathways see Supporting information 1 and 2. (d) PC analysis of the vastus lateralis proteome in older and younger adults before and after resistance training. PC1, 42.58%; PC2, 10.90%. FDR, false discovery rate; PC, principal component.

Pathways related to 13 overarching themes (Figure 1a) comprising 66 ‘pathways’ (Figure 1b) were augmented by RT in younger VL (Figure 1a,b). Ten themes were augmented by RT in older VL (Figure 1a). These themes comprised 43 Reactome ‘pathways’. The diversity of response of the older VL proteome to RT is evident in Figure 1b,c; details regarding pathways detailed in these panels can be found in Supporting information 1 and 2.

PCA was used to separate the VL proteomes by age and pre‐/post‐training status. Three‐component PCA (PC1, 42.58%; PC2, 10.90%; PC3, 6.34%) clearly separated younger and older proteomes, with pre–post training separations being less evident (Figure 1d). Therefore, our remaining analysis attempts to further characterize this age‐associated separation between the cohorts.

### 3.4 Effect of age and 8 weeks’ resistance training on skeletal muscle protein expression

**FIGURE 2:**
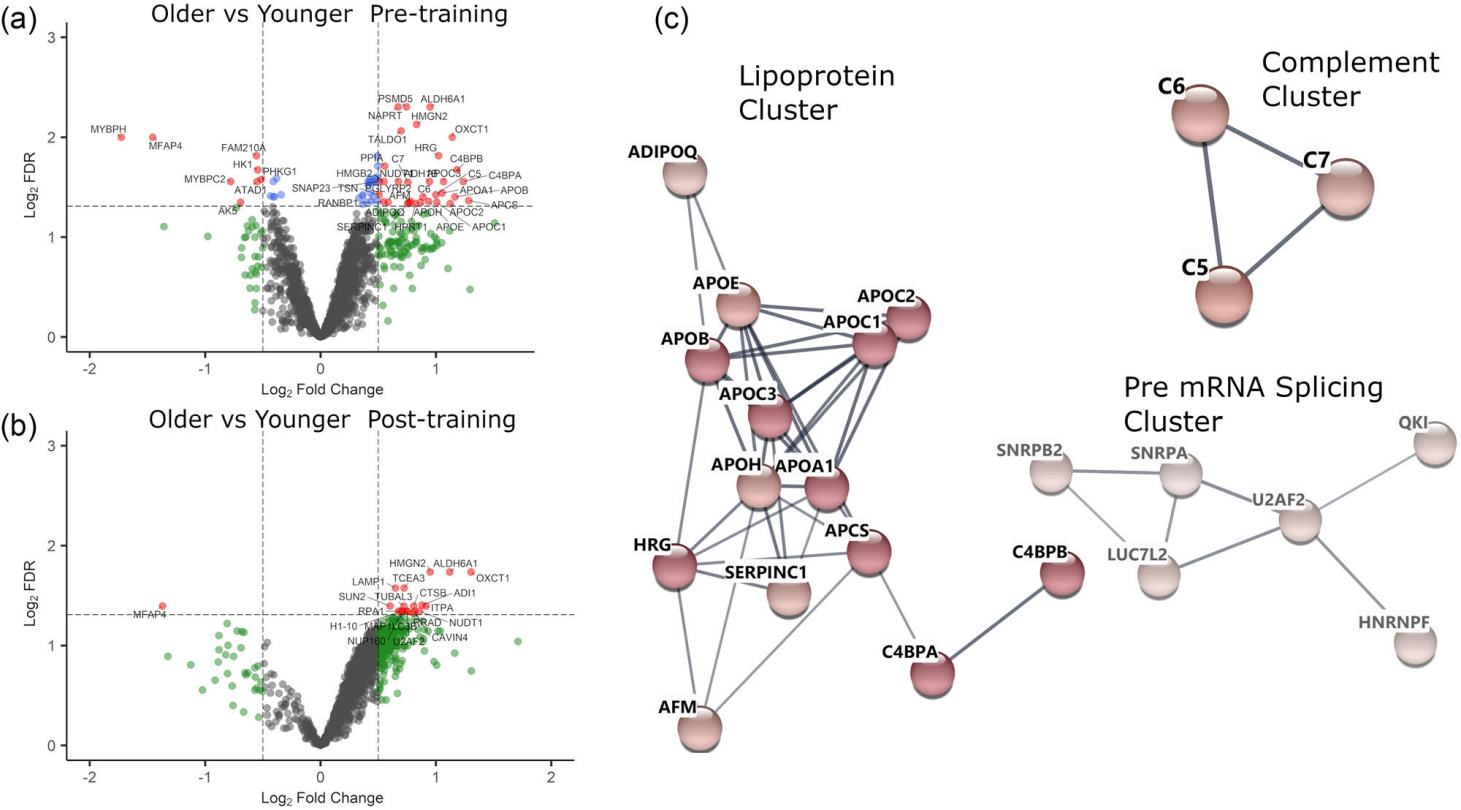
(a, b) Volcano plot of differential skeletal muscle protein expression in the skeletal muscle of older women (*n* = 8) compared to younger (*n* = 7) controls prior to (a) and following (b) resistance training. Red: log_2_FC < −0.5 or < 0.5 and log_2_FDR > 1.3 (equivalent to an adjusted *P* = 0.0490); blue: log_2_FC between −0.5 and 0.5, log_2_FDR > 1.3; green: log_2_FC < −0.5 or < 0.5, log_2_FDR < 1.3; grey: log_2_FC between −0.5 and 0.5, log_2_FDR < 1.3. (c) Visualisation of skeletal muscle protein functional interaction networks. Proteins that were differentially expressed between age cohorts prior to resistance training were subjected to Markov clustering. Log_2_FCs of skeletal muscle protein expression between young (*n* = 7) and older (*n* = 8) women were mapped to nodes using a blue–white–red gradient. The full STRING network was used with a high confidence threshold of 0.7 for edge representation. Edge thickness represents probability that a predicted link exists between two protein nodes. FC, fold change; FDR, false discovery rate.

Prior to RT, 70 individual proteins (17 downregulated, 53 upregulated) were differentially expressed in the skeletal muscle of older women compared to younger controls. Expression of myosin‐binding protein H (Q13203) and microfibril‐associated glycoprotein 4 (P55083) was lower in older skeletal muscle. Expression of apolipoprotein B‐100 (P04114), C4b‐binding protein β chain (P2085), C4b‐binding protein α chain (P4003) and serum amyloid P‐component (P02743) was higher in older VL (Figure 2a, Supporting information 3).

Over‐representation analyses indicated that GO biological process terms related to the immune system were enriched in older skeletal muscle—innate immune response (19% proteins involved in term, FDR < 0.0001), complement activation, and classical pathway (8.2%, FDR = 0.029). KEGG complement and coagulation cascade pathways were also enriched (8.2%, FDR = 0.044). KEGG cholesterol metabolism pathways were enriched (9.6%, FDR < 0.0001). GO biological process terms related to cholesterol metabolism (cholesterol efflux, phospholipid efflux, chylomicron clearance, lipoprotein metabolic process, triglyceride homeostasis, high‐density lipoprotein particle remodelling, reverse cholesterol transport) were also significantly enriched in older skeletal muscle. These observations are corroborated by Markov clustering of protein expression data (Figure 2c)

Following RT, 20 individual proteins (2 downregulated, 18 upregulated) were differentially expressed in the skeletal muscle of older women compared to younger controls (Figure 2b, Supporting information 4). Nine of these proteins were also differentially expressed in the skeletal muscle of older women prior to RT. Expression of succinyl‐CoA:3‐ketoacid coenzyme A transferase 1 (P55809), methylmalonate semialdehyde (Q02252) and non‐histone chromosomal protein HMG‐17 (P05204) were increased in older women compared to their younger counterparts following RT. Notably, expression of microfibril‐associated glycoprotein 4 (P55083) remained significantly lower in older women following RT. Expression of splicing factor U2AF 65 kDa subunit (P26368), SUN domain‐containing protein 2 (Q9UH99), oxidized purine nucleoside triphosphate hydrolase (P36639), and histone H1.10 (Q92522) remained higher in older skeletal muscle following RT.

Over‐representation analysis of these 20 proteins did not reveal any significantly enriched processes or terms. Over‐expression analyses rely upon a limited panel of differentially expressed proteins to highlight pathways with enriched terms given a particular gene/protein list. These analyses do not consider quantitative data from all proteins that have been measured in a sample. Therefore, we conducted differential expression analyses of our data to gain further insights.

### 3.5 Proteomic pathways differentially regulated by older age in female skeletal muscle pre‐ and post‐training

**FIGURE 3:**
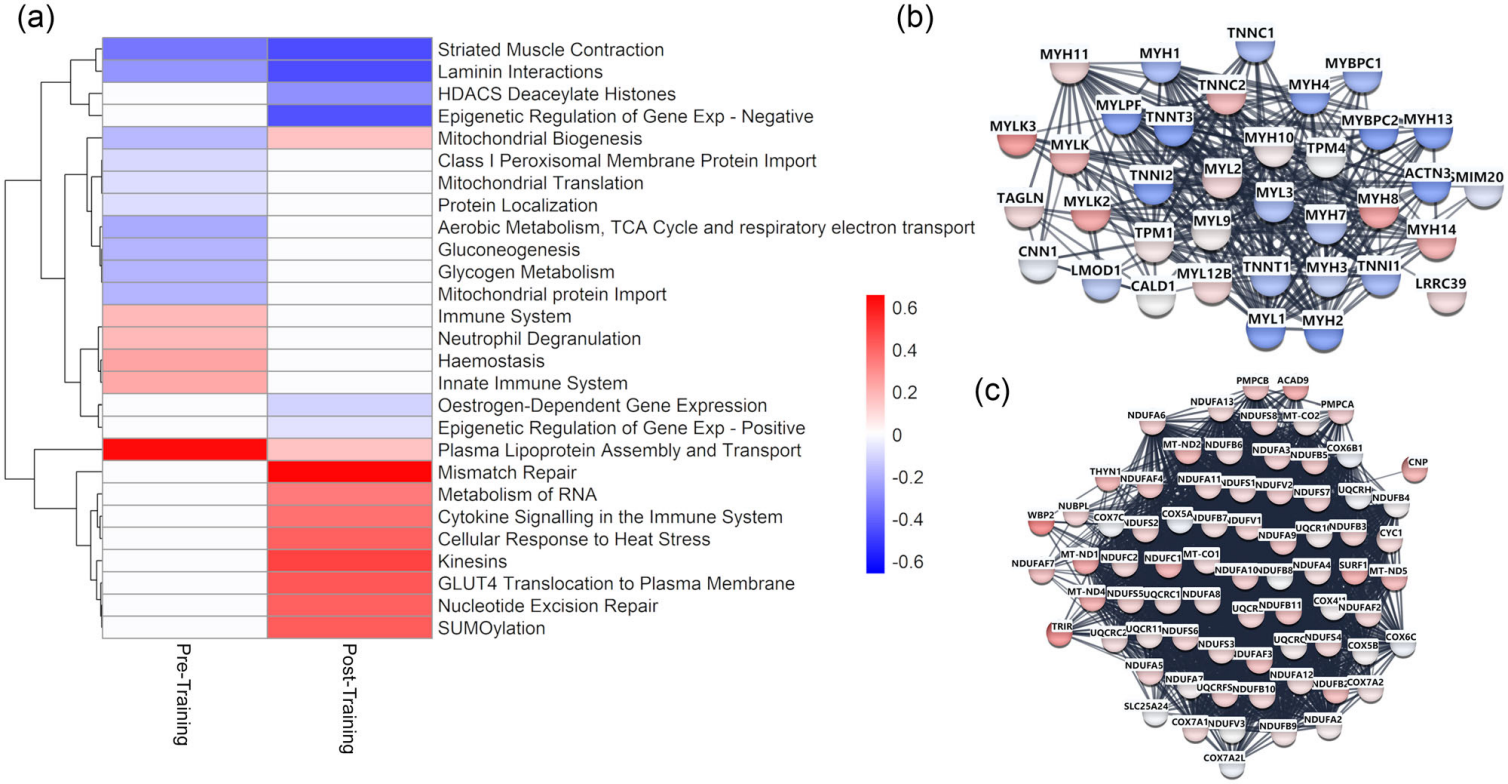
Proteomic themes differentially regulated by older age in female skeletal muscle pre‐ and post‐training. (a) Reactome pathway themes that significantly differ between younger and older women pre‐ and/or post‐resistance training. A blue–white–red gradient was styled using the log_2_fold change value for each theme compared to younger women. White: non‐significant difference between age cohorts (false discovery rate > 0.05). (b,c) Visualisation of selected skeletal muscle protein networks (muscle contractile proteins (b) and mitochondrial proteins (c)) altered by age following resistance training. Log_2_fold changes of skeletal muscle protein expression between young (*n* = 7) and older (*n* = 8) women were mapped to nodes using a blue–white–red gradient. The full STRING network was used with a high confidence threshold of 0.7 for edge representation. Edge thickness represents probability that a predicted link exists between two protein nodes.

Prior to RT, differential protein expression analyses showed that older women had lower expression of proteins in pathways related to aerobic metabolism (log_2_FC −0.21, FDR < 0.0001), glycogen metabolism (log_2_FC −0.19, FDR = 0.00193) and gluconeogenesis (log_2_FC −0.18, FDR = 0.00101) compared to their younger counterparts. Further, there was a decrease in proteins related to skeletal muscle contraction (log_2_FC −0.34, FDR < 0.0001), laminin interactions (log_2_FC −0.26, FDR = 0.0207), and mitochondria (e.g., mitochondrial biogenesis; log_2_FC −0.17, FDR  < 0.0001) (Figure 3, Supporting information 3). Older women displayed an increased expression of proteins related to the immune system (log_2_FC 0.19, FDR = 0.001480), haemostasis (log_2_FC 0.24, FDR = 0.0239) and lipoprotein assembly (log_2_FC 0.64, FDR = 0.00862).

Following 8 weeks of lower limb RT, older women exhibited lower VL expression of proteins related to skeletal muscle contraction (log_2_FC −0.45, FDR < 0.0001), oestrogen‐dependent gene expression (log_2_FC −0.11, FDR = 0.00172), epigenetic regulation of gene expression (log_2_FC −0.06, FDR = 0.0300), and HDAC deacetylation of histones (log_2_FC −0.28, FDR = 0.0256) compared to younger controls (Figure 3, Supporting information 4). Following RT, older women had higher expression of VL proteins related to cytokine signalling in the immune system (log_2_FC 0.38, FDR = 0.0277), metabolism of RNA (log_2_FC 0.35, FDR = 0.0160), SUMOylation (log_2_FC 0.43, FDR = 0.0312), cellular response to heat stress (log_2_FC 0.41, FDR = 0.0180), GLUT4 translocation to plasma membrane (log_2_FC 0.44, FDR = 0.0114), kinesins (log_2_FC 0.49, FDR = 0.0320) and mitochondrial biogenesis (log_2_FC 0.16, FDR = 0.00200) (Figure 3). STRING visualisations of the protein network with Markov clustering identified clusters that corroborate differential pathway analyses (Figure 3).

### 3.6 Relationship between age‐associated skeletal muscle proteins and neuromuscular function

**FIGURE 4:**
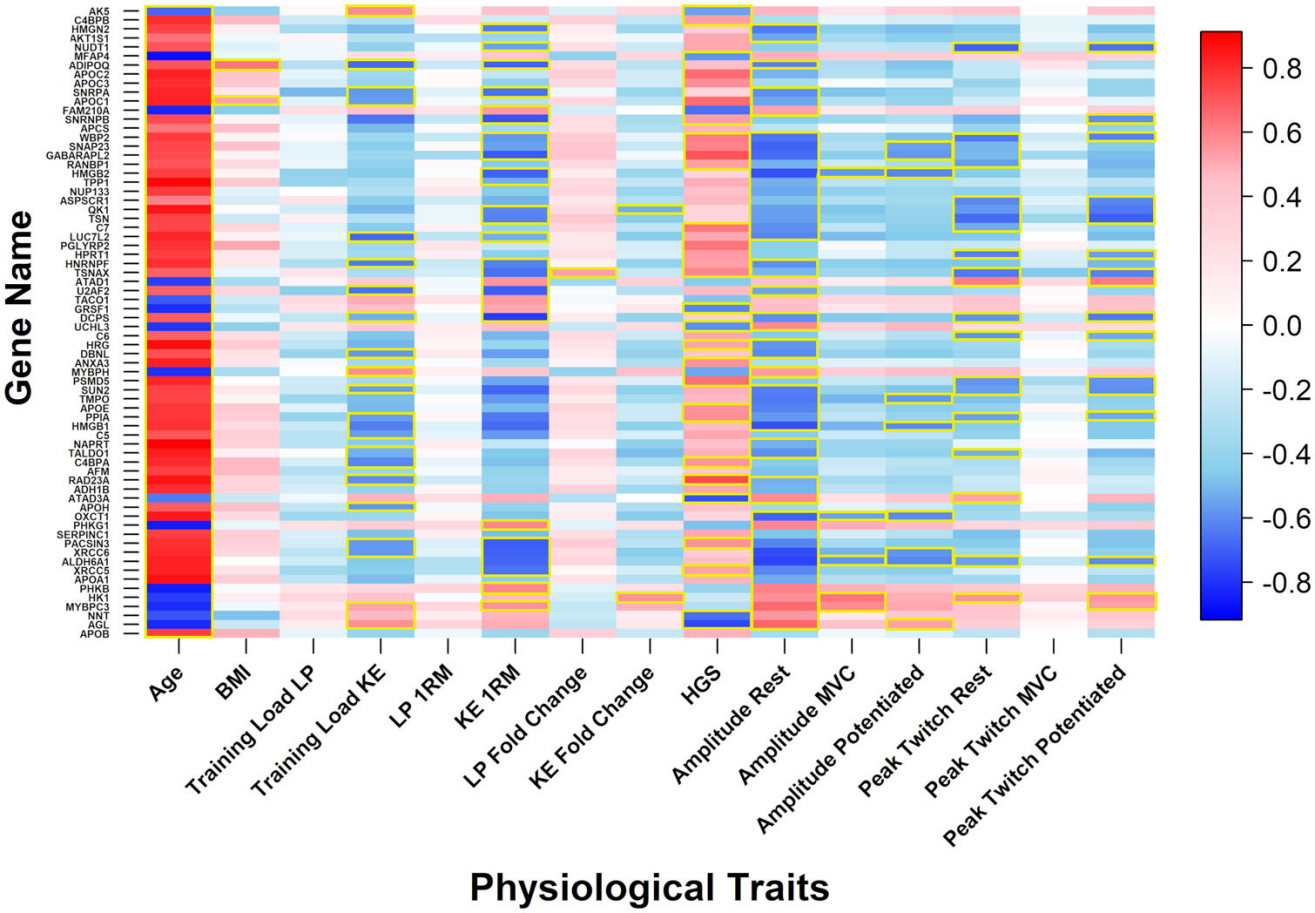
Pearson correlations between proteins that were differentially expressed between age groups prior to training and physiological traits prior to exercise training. Data from older (*n* = 8) and younger (*n* = 7) women were used to calculate these correlations. KE and LP training loads were calculated for each participant from weekly total repetitions completed multiplied by total weight lifted (kg) for each of the 8 training weeks. A trapezoid area under the curve was used to calculate a training load value for each participant. A blue–white–red gradient indicates Pearson value. Yellow box: *P* < 0.05 for Pearson correlation; ‘Amplitude’ refers to M‐wave amplitude. 1RM, one repetition maximum; KE, knee extension; MVC, maximum voluntary contraction; LP, leg press.

Pearson correlations were explored between proteins that were differentially expressed between age‐groups pre‐training and their functional outcomes. Age‐related changes in individual VL protein expression were consistently associated with poorer leg strength/NMF (Figure 4). However, such changes were often associated with increased HGS.

**FIGURE 5:**
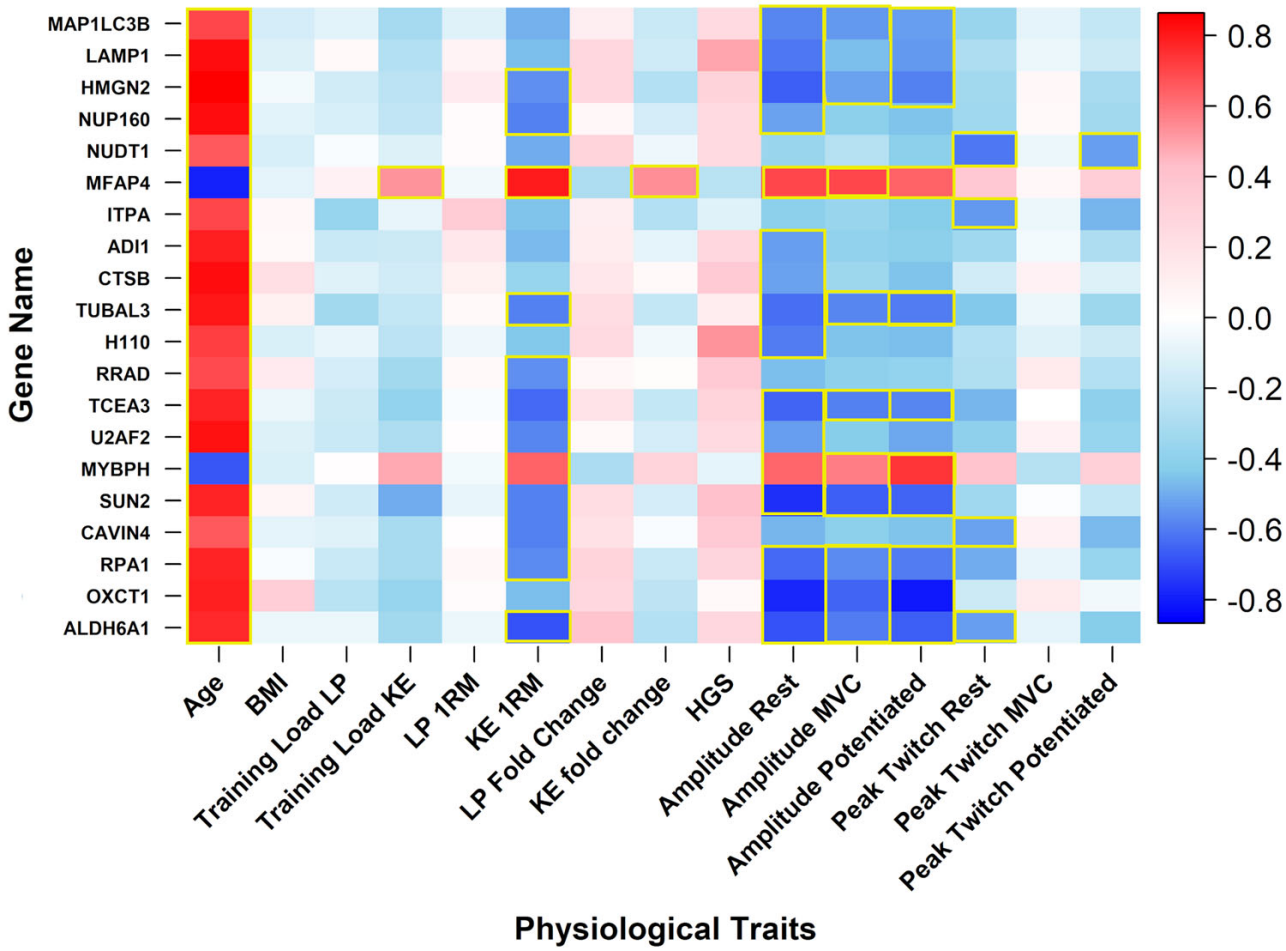
Pearson correlations between proteins that were differentially expressed between age groups following training and physiological traits following exercise training. Data from older (*n* = 8) and younger (*n* = 7) women were used to calculate these correlations. KE and LP training loads were calculated for each participant from weekly total repetitions completed multiplied by total weight lifted (kg) for each of the 8 training weeks. A trapezoid area under the curve was used to calculate a training load value for each participant. A blue–white–red gradient indicates Pearson value. Yellow box: *P* < 0.05 for Pearson correlation; ‘Amplitude’ refers to M‐wave amplitude. 1RM, one repetition maximum; KE, knee extension; LP, leg press; MVC, maximum voluntary contraction.

Pearson correlations were also explored between proteins that were differentially expressed between age cohorts post‐training and post‐training functional outcomes. The effect of age on individual protein expression was—as with pre‐exercise data—consistently associated with poorer strength/NMF (Figure 5).

WGCNA identified 14 protein modules, with five modules having a significant association with age (*P* < 0.01). No protein modules displayed a significant relationship with time (pre‐ vs. post‐exercise training). Notably, expression of proteins in modules 3 and 5 was positively correlated with age (module 3: *r* = 0.9, *P* < 0.00001; module 5: *r* = 0.79, *P* < 0.00001). These modules contained a broad range of proteins involved in glycogen metabolism, mitochondrial biogenesis and energy metabolism, cellular signalling, gene regulation, and protein folding and chaperone activities. These modules were also negatively correlated with KE training load (module 3: *r* = −0.37, *P* = 0.0002; module 5: not significant), KE 1RM age (module 3: *r* = −0.12, *P* = 0.001; module 5: *r* = −0.05, *P* < 0.00001) and measures of excitability at rest, during contraction and potentiated (e.g., VL EMG M‐wave amplitude during MVC: module 3: *r* = −0.52, *P* = 0.003; module 5: *r* = −0.37, *P* = 0.04) (Supporting information 5, 6, 7).

## 4 DISCUSSION

Here, we describe a quantitative characterisation of the aged human skeletal muscle proteome both before and after a period of RT. Further, we present a detailed neuromuscular characterisation of participants, which has allowed us to identify correlations between differentially expressed proteins and physiological traits.

In older women, the pre‐training proteome showed significantly lower expression of proteins related to skeletal muscle contraction, laminin interactions, mitochondrial biogenesis, aerobic metabolism, glycogen metabolism and gluconeogenesis compared to that of younger counterparts. Further, there was reduced expression of proteins related to skeletal muscle contraction in older women. Older women displayed an increased expression of proteins related to the immune system, haemostasis assembly, DNA mismatch repair, and RNA polymerase III transcription. Post‐training, the older skeletal muscle proteome was notable in the persistence of this lower expression of proteins related to skeletal muscle contraction—this despite similar training‐induced percentage increases in LP and KE 1RM compared to younger controls. Following RT, older women had higher expression of VL proteins related to biogenesis compared to the younger cohort, a reversal of the phenomenon observed pre‐training. However, expression of proteins related to aspects of aerobic metabolism no longer differed between age cohorts following RT. Proteins related to cytokine signalling and DNA repair were also enriched in the older cohort. Pre‐training, 70 individual proteins were differentially expressed between age cohorts. Post‐training, 20 individual proteins were differentially expressed between the cohorts. At the VL expression levels observed in older individuals, these proteins consistently predicted poorer leg strength/NMF. Conversely, the VL expression level of these proteins in older individuals was often associated with greater HGS.

### 4.1 Contractile and structural proteins

Proteins related to skeletal muscle contraction were increased by RT in both age cohorts. However, their expression was lower in older skeletal muscle compared to that of younger women both before and after RT. Our pre‐training observations are largely in agreement with previous proteomic studies (Brocca et al., 2017; Deane et al., 2023; Gueugneau et al., 2014; Staunton et al., 2012; Théron et al., 2014). Our findings suggest that short to medium term RT can increase, but not completely restore VL contractile protein content in older age. Previous work which adjusted for self‐reported physical activity levels has suggested that longer‐term activity may also not normalise such protein expression (Ubaida‐Mohien, Lyashkov et al., 2019). One recent study has examined the effect of habitual (15 years or more) RT on the skeletal muscle proteome, compared to that of young untrained controls. This study suggested limited effects of such training on the skeletal muscle proteome. However, these participants were all male and relatively young (34–53 years) and as such unlikely to yet have accrued substantial sarcopenic changes. No study has yet conducted comprehensive skeletal muscle proteomic profiling in lifelong athletes. This may offer the best indication of the extent to which aspects of the skeletal muscle contractile apparatus are diminished by age per se. This may in turn allow for more targeted interventions (e.g., pharmacological) that could act as adjuncts to lifelong physical activity in arresting sarcopenia.

Further, proteins related to the extracellular matrix (ECM)/laminin were diminished in older skeletal muscle both before and after RT. In Reactome's peer‐reviewed pathway database, this theme encompasses laminin–collagen and laminin–integrin interactions within the ECM. The ECM is increasingly recognised as important for the effective transmission of mechanical signals to the intracellular environment, for downstream adaptation to mechanical forces, and for being able to directly influence myofibre contractility (Azizi et al., 2008; Hastings et al., 2019). Our findings suggest that short–medium term RT interventions do not result in a ‘younger’ ECM environment in older skeletal muscle. It is unclear whether this would favour effective contractile function in older age. The effects of long‐term exercise training on human skeletal muscle ECM remodelling remain unstudied. Our observations are also notable as the ECM has thus far been understudied in skeletal muscle proteomic studies, with some choosing to focus on sarcoplasmic fractions, rather than total extracts (Deane et al., 2023). Each protein extraction method has relative advantages and disadvantages (Dowling et al., 2023). The prominence of ECM changes in aged skeletal muscle indicates that proteomic studies should consider this (in conjunction with their research question) when choosing a lysate preparation method.

Notably, myosin‐binding protein H (Q13203) and microfibril‐associated glycoprotein 4 (P55083) were prominently diminished in older skeletal muscle, confirming the observations of (Ubaida‐Mohien, Lyashkov et al., 2019). We have now shown that the skeletal muscle content of these individual proteins is not normalised by RT. Additionally, following RT, skeletal muscle content of these proteins showed a significant positive correlation with KE training load, KE 1RM and KE strength accrual (fold change from pre‐training 1RM), and measures of skeletal muscle excitability. Of these, KE training load and KE strength accrual were not independently correlated with age. Microfibril‐associated glycoprotein 4 is an ECM protein that can bind to collagen and elastin (Kanaan et al., 2022). Its function in skeletal muscle is not well characterised. Myosin‐binding protein H appears to play a key role in muscle structure, function and integrity, although its precise function is as yet unclear. It is underexpressed in the pelvic skeletal muscle of those at risk of pelvic organ prolapse (Hundley et al., 2006), and is overexpressed in the skeletal muscle of those with amyotrophic lateral sclerosis (Conti et al., 2014) and myotonic dystrophy type 1 (Aoussim et al., 2023). The dysregulated expression of myosin‐binding protein H may play a causative role in some or all of these (patho)physiological states, but so too may be a biomarker of ageing in skeletal muscle. Indeed, age per se is an important confounder in considering the causative importance of such proteins with functional outcomes in our dataset. Nonetheless, some functional/causative role for such proteins is suggested by their correlation with outcomes that did not have a statistical relationship with age, for example, myosin‐binding protein H expression was positively correlated with the age‐independent KE training load.

While the VL expression levels of myosin‐binding protein H and microfibril‐associated glycoprotein 4 observed in older individuals predicted poorer leg strength/NMF, their VL expression prior to training predicted greater HGS. Indeed, the VL expression of many proteins that were differentially expressed between the cohorts pre‐training predicted poorer leg strength/NMF but greater absolute HGS. HGS was not independently associated with age in our dataset, an observation that is in contrast to the literature (Bohannon, 2012; Strandkvist et al., 2021). This is likely explained by the narrow age ranges and small sample sizes in each of our cohorts. These observations suggest complex age‐ and muscle‐specific mechanisms may exist in the relationship between skeletal muscle proteomes and muscular function. The correlations between individual protein content and functional outcomes presented here are merely a signpost towards a mechanistic understanding of skeletal muscle function in older age. Some of these relationships may be causative. Many are likely subject to multiple confounders. It is evident from our data that the molecular milieu in one muscle (VL) does not appear to predict function in another muscle group (forearm, HGS). The human forearm flexors are less well studied than the VL, but these flexors appear to contain a substantially higher proportion of type II fibres (cf. 45% vs. 65%) into older age (Moore et al., 2021; Pollock et al., 2018). Indeed, it has emerged that the skeletal muscle proteome can be effectively sub‐divided by fibre type (Deshmukh et al., 2021; Murgia et al., 2017). There also exits large inter‐muscle variability in motor unit innervation number, with hand muscles displaying lower innervation number than large muscles such as VL. This may play a role in the complex interplay between molecular and functional outcomes (Duchateau & Enoka, 2022). Future research should endeavour to discover unifying molecular mechanisms that determine skeletal muscle function in all muscle groups, but so too muscle/muscle group specific mechanisms.

### 4.2 Mitochondria

Prior to training we observed that proteins related to mitochondrial biogenesis and aerobic fuel metabolism were lower in older women compared to their younger counterparts. Studies using targeted investigations of gene transcripts and proteins have long‐established an age‐related reduction in pathways related to mitochondrial biogenesis and aerobic fuel metabolism (Jaleel et al., 2008; Konopka & Sreekumaran Nair, 2013; Rooyackers et al., 1996; Short et al., 2005). This has been confirmed by more recent proteomic studies (Deane et al., 2023; Ubaida‐Mohien, Lyashkov et al., 2019). Further, it is established that ageing is associated with reduced skeletal muscle glycogen storage (Dubé et al., 2016), corroborating our findings of reduced proteins related to glycogen metabolism and gluconeogenesis. Interestingly, it is also known that such glycogen stores can be increased with physical training in older adults (Dubé et al., 2016). Indeed, we observed a significant increase in proteins associated with glycogen metabolism pathways following RT in older women. As a result, there were no post‐training differences in glycogen pathway protein enrichment between young and older individuals.

Following RT, differential protein expression analyses indicated an increase in proteins related to mitochondrial biogenesis in older women, compared to the young. This training‐induced increase in such proteins in older women was confirmed via pre–post differential pathway analyses. Decades of RT literature have performed targeted, if limited, molecular investigations in skeletal muscle. The literature has long suggested that older adults may be more likely to derive mitochondrial biogenesis benefits from RT interventions than younger adults; this has remained controversial in some quarters (Groennebaek & Vissing, 2017). Our data now strongly corroborate this notion. RT alone does improve cardiorespiratory fitness in older adults (Smart et al., 2022), although its combination with aerobic training appears to be superior (Khalafi et al., 2022).

Expression of the translational activator of cytochrome *c* oxidase 1 was decreased with age prior to training, yet this protein was positively correlated with KE baseline 1RM. Translational activator of cytochrome *c* oxidase 1 is thought to be required for cytochrome *c* oxidase 1 translation (Richman et al., 2016) and thus plays a crucial role in the formation of the final enzyme of the electron transport chain. It is well known that ageing is associated with a reduced capacity of mitochondria to generate ATP and many studies have linked this change to reduced muscle power in endurance exercise scenarios (Conley, 2016). However, in 1RM testing with generous rest intervals, all energetic needs can likely be met by phosphagen stores that have sufficient time to regenerate between attempts. Therefore, it seems likely that this protein is a biomarker of broader skeletal muscle health and therefore strength.

Baseline hexokinase I expression was decreased with age; its expression positively correlated with subsequent KE strength accrual due to training. Hexokinase I is less well studied than the insulin‐responsive hexokinase II. It is thought that hexokinase I binding and dissociation from the mitochondrial membrane might act as a metabolic switch between catabolism and anabolism (De Jesus et al., 2022). Our data suggest that a catabolic/anabolic switch may indeed be of functional relevance, promoting greater strength gains in response to a training stimulus. This requires further exploration.

### 4.3 Immune system

Proteins related to skeletal muscle immune/inflammation pathways were significantly enriched in older skeletal muscle prior to training. Our pre‐training observations are consistent with the previous high‐quality study of untrained aged skeletal muscle (Ubaida‐Mohien, Gonzalez‐Freire et al. 2019). We too observed a prominent age‐related VL enrichment of proteins related to the complement pathway (complement C5, C7, C6, C4b‐binding protein α chain) and an increased acute phase protein serum amyloid P‐component (Ubaida‐Mohien, Gonzalez‐Freire et al., 2019). In our study, these complement proteins were negatively correlated with some measure(s) of skeletal muscle function prior to training. Limited evidence has suggested that complement may play a role in skeletal muscle regeneration (Rouaud et al., 2017), and circulating complement pathway components may be biomarkers for sarcopenia (Nakamura et al., 2024). Complement activation is thought to contribute to the loss of muscle acetylcholine receptors (AChR) in myasthenia gravis, leading to impaired synaptic transmission at the neuromuscular junction (Howard, 2018). AChRs are also lost in the development of sarcopenia (Moreira‐Pais et al., 2022). Notably, RT reduced the VL expression of proteins associated with the immune system in older women and normalized the post‐training expression of proteins in these pathways between age cohorts. It appears that if such immune pathways are indeed molecular determinants of skeletal muscle function in older age, then they are amenable to modification via RT; this warrants further study.

Post‐training, cytokine signalling pathways were enriched in older skeletal muscle, although individual proteins related to inflammation were not found to be differentially expressed. It has been suggested that there is an optimal myokine milieu for skeletal muscle adaptation to a training stimulus, but it remains unclear as to what this milieu is and indeed if it differs between individuals and age groups (Cornish et al., 2020). Twelve weeks’ RT has previously been suggested to normalise skeletal muscle cytokine concentrations between young and older individuals, both before and after an acute training stimulus (Della Gatta et al., 2014). However, that study examined a limited panel of cytokines via ELISA of skeletal muscle lysates. Wider evidence is sparse, and previous proteomics studies of RT effects on skeletal muscle may not have been capable of detecting subtle changes in skeletal muscle inflammation, that is, one of these studies detected only 164 proteins for downstream bioinformatics (Deane et al., 2023) and another appeared to quantify only mitochondrial proteins (Robinson et al., 2017). Neither approach allowed the power of differential protein expression analyses to be fully exploited.

### 4.4 RNA metabolism

The ‘metabolism of RNA’ pathway encompasses a multitude of processes that lead to mature, functional RNA production. Pathways related to these processes were enriched in older skeletal muscle following RT. In particular, proteins in pathways related to processing of pre‐mRNA were enriched. Rodent research has suggested that pre‐mRNA processing is impaired in older satellite cells (Malatesta et al., 2010) and it appears that aged skeletal muscles do not export mature mRNA from the nucleus at the same rate as younger counterparts (Cisterna & Malatesta, 2024). Rodent evidence has suggested that exercise may partially reverse this (Malatesta et al., 2010), a notion that our data now support. Further, a recent powerful ‘omics’ meta‐analysis found that exercise training can alter the aged epigenome and transcriptome, moving these towards a ‘younger’ state (Voisin et al., 2024). It has also been established that—downstream of mature mRNA production—ribosomal biogenesis is impaired in older adults undergoing RT compared to the young (Brook et al., 2016, 2019). Here, we have described a post‐training decrease in proteins related to overall (positive and negative) epigenetic regulation of ribosomal gene expression in older skeletal muscle. This may imply a state of reduced turnover, which warrants exploration. Proteins related to these transcriptional and translational events (i.e., non‐histone chromosomal protein HMG‐17 (P05204), splicing factor U2AF 65 kDa subunit (P26368), transcription elongation factor A protein 3 (O75764), histone H1.10 (Q92522)) were often negatively correlated with KE 1RM and measures of peak twitch and excitability. These outcomes also displayed confounding correlations with age.

### 4.5 Lipoprotein assembly

In older participants, we note a pre‐training enrichment of skeletal muscle proteins related to plasma lipoprotein assembly. Reactome, DAVID and STRING analyses indicated that apolipoproteins were prominent. Increased skeletal muscle APOA1 has been associated with improved glucose uptake and mitochondrial function (Giacona et al., 2024; Tang et al., 2019). Older participants in this study were generally fit and physically active prior to the addition of RT and our findings may reflect this. However, the enrichment of plasma lipoprotein assembly proteins over those found in younger adults requires further investigation.

### 4.6 Limitations

This study has several important limitations. First, participants were taking part in a wider herbal supplementation study, and as such consumed a placebo (magnesium stearate 1100 mg/day) for the duration of the training programme. The WHO Expert Committee on Food Additives estimates the dietary exposure to magnesium stearate from bakery products, confectionary and food supplements as c. 83 mg/kg body weight per day. The addition of two 550 mg placebo capsules per day would increase typical magnesium stearate intake by 22% in a 60 kg individual. We consider this unlikely to have had a meaningful effect on the skeletal muscle proteome described here. Tests of NMF were conducted immediately prior to VL biopsy. Therefore, the skeletal muscle proteome described is representative of that following a bout of moderate RT. Translational changes to the proteome are not thought to be prominent over such a short time frame. The differential proteomic findings reflect the entire tissue composition rather than just those in myofibers. While this may introduce variability due to age‐related differences in cell composition, it also provides a physiologically relevant view of the tissue, making it a consideration rather than a strict limitation of the study. We did not perform our analyses on different myofibre isoform types. Previous literature suggests that such an approach can identify fibre‐type specific proteomic shifts (Deshmukh et al., 2021; Murgia et al., 2017). This study did not calculate an a priori sample size; rather it was conceived due to the prominence of age‐related changes in the proteome when data from the original study were analysed. Indeed, hypothesis‐free proteomic pathway analyses are not readily conducive to traditional sample size calculations. Due to the nature of the original study, these data are from female participants only and therefore redresses the sex‐imbalances in the literature. However, larger studies of mixed‐sex cohorts would be desirable. We note that PCA analysis revealed that three post‐training samples from the younger cohort notably diverged from their counterparts for PC1. No methodological or quality control issue were identified for these data. This phenomenon likely represents normal biological variability in training response in young skeletal muscle. Similarly, while our normalisation procedures were carefully conducted to minimise technical variation, the volcano plot in Figure 2b shows a biologically plausible skew towards upregulated proteins in older adults following RT. This analysis only captures the proteins that were measurable with our proteomics approach. Many proteins in skeletal muscle tissue may not have been detected, and only 20 proteins reached statistical significance. Therefore, the observed skew should not be extrapolated to reflect an imbalance in total protein load across all proteins in the tissue. We did not apply multiple comparison corrections to the Pearson correlation analyses between individual proteins and physiological traits. This was a deliberate decision, as the analysis was exploratory and aimed at identifying potential relationships between biologically linked variables. Applying stringent corrections in this context could obscure meaningful insights, especially since the variables and proteins included were already pre‐selected based on prior filtering and experimental decisions. This approach aligns with similar studies in the field (for example Ubaida‐Mohien, Lyashkov, et al., 2019) and maintains the exploratory value of the analysis. In considering the importance of these data, it should be noted that they are not from older sarcopenic women; rather they represent the characterisation of a healthy older cohort. However, the decline in skeletal muscle quality with age exists as a spectrum rather than being binary. These insights are therefore relevant to pre‐sarcopenic and sarcopenic skeletal muscle. Indeed, we found no difference in LP 1RM between age cohorts at baseline and a greater relative increase in LP 1RM in older participants following training. Despite efforts to control for factors such as prior RT experience, learning effects and participant range of motion, these factors confound these observations in a small previously untrained population. We also note that the older cohort displayed higher HGS at baseline; while unexpected, this is not an implausible statistical outcome when comparing two small cohorts.

### 4.7 Conclusion

We have described the differential effect of RT on the skeletal muscle proteome in older and younger adults. Many of our post‐training observations greatly expand what is known about the effect of RT on the skeletal muscle proteome. Post‐training, the older skeletal muscle proteome was notable in the persistence of lower expression of proteins related to skeletal muscle contraction. This may indicate that these proteins are suitable for targeted pharmacological interventions that could act as adjuncts to physical activity in arresting sarcopenia. Our data corroborate the notion that older adults derive more prominent mitochondrial biogenesis benefits from RT interventions than younger adults.

We have identified individual proteins within this aged proteome that consistently predict poorer leg strength/NMF. However, VL expression of these proteins in older individuals was often associated with greater HGS; this is a novel and important observation. Future research should endeavour to dissect these complex relationships. It may be that unifying molecular mechanisms of skeletal muscle responses to RT can be delineated. Our data suggest that there also likely exist muscle‐specific mechanisms to be unravelled.

## AUTHOR CONTRIBUTIONS

Mary O'Leary and Joanna Bowtell conceived and planned the experiments. Mary O'Leary, Elsa Greed, Jack Pritchard, Lauren Struszczak and Esra Bozbaş carried out the experiments. Mary O'Leary analysed the results. Mary O'Leary and Joanna Bowtell contributed to the interpretation of the results. Mary O'Leary led the writing of the manuscript. All authors provided critical feedback and helped shape the final manuscript, including revising it critically for important intellectual content. All authors have read and approved the final version of this manuscript and agree to be accountable for all aspects of the work in ensuring that questions related to the accuracy or integrity of any part of the work are appropriately investigated and resolved. All persons designated as authors qualify for authorship, and all those who qualify for authorship are listed.

## CONFLICT OF INTEREST

The authors declare no competing interests.

## Supporting information




**Supporting information 1**. Reactome results CAMERA analysis; pre‐post change in younger group.

**Supporting information 2**. Reactome results CAMERA analysis; pre‐post change in older group.

**Supporting information 3**. Reactome results CAMERA analysis; older vs younger group pre‐training.

**Supporting information 4**. Reactome results CAMERA analysis; older vs younger group post‐training.

**Supporting information 5**. WGCNA module vs trait correlation coefficients.

**Supporting information 6**. WGCNA module vs trait correlation p values.

**Supporting information 7**. WGCNA module key.

## Data Availability

The datasets used and/or analysed during the current study are available in PRIDE at https://www.ebi.ac.uk/pride/archive/projects/PXD056507/. The code used to implement the WGCNA analysis is available from https://github.com/mfoleary/PUK_Proteomics
